# Fine scale behaviour and time-budget in the cryptic ectotherm European pond turtle *Emys orbicularis*

**DOI:** 10.1371/journal.pone.0256549

**Published:** 2021-10-15

**Authors:** Théo Marchand, Anne-Sophie Le Gal, Jean-Yves Georges

**Affiliations:** 1 Université de Strasbourg, CNRS, IPHC, UMR 7178, Strasbourg, France; 2 Research Station Petite Camargue Alsacienne, Saint-Louis, France; State Museum of Natural History, GERMANY

## Abstract

For ectotherms, behaviour and associated energetic costs are directly related to thermal conditions. In the present context of global change, estimating time-budget for these species is relevant to assess and predict their capacity to adapt to near future. We tested the hypothesis that in ectotherms where reproduction is highly energy consuming, energy expenditure should vary throughout the breeding season with a maximum around nesting events. To test this hypothesis, we assessed the fine-scale behaviour, time-budget and estimated energetic costs in eight adult female European pond turtles *Emys orbicularis* equipped with data-loggers recording ambient temperature, pressure, light and the animals’ 3-axis acceleration. Deployments occurred over four months throughout the nesting season 2017 in semi-natural captive conditions in Alsace, France. All study turtles showed a clear daily pattern over the 24h cycle, with four distinct phases (referred to as Night, Morning, Midday and Evening), associated with different behaviours and activity levels. Before oviposition, turtles were mostly active during Morning, and activity was positively driven by ambient temperature. Activity levels doubled during the nesting period, mostly due to the increased activity in the Evening, when nesting events occurred. Throughout the active season, basking occurrence at Midday was related to air temperature but cloud coverage was an even more important factor. Our results are a first step in predicting the seasonal time and energy budgets of the European pond turtle, and demonstrate the usefulness of animal-borne accelerometers to study free living freshwater turtles over extended periods of time.

## Introduction

Human induced rapid environmental changes [1] include climate change, habitat fragmentation and loss, pollution, species invasions and extinctions. These changes and especially global warming are expected to have a more direct impact on ectotherms than on endotherms, as their physiology acutely depends on ambient temperature [2]. Temperature changes have been reported to have diverse effects on both behaviour and physiology of insects, with contrasted consequences on populations, depending on the species and conditions (e.g. [3, 4]). Consequences for fish have been most investigated, with changes in water temperature affecting heart rate, activity and thermoregulation (e.g. [5]). In this context, Sauropsids (reptiles) have been less studied (but see [6, 7]) and more particularly marine and freshwater turtles. This latter gap in freshwater turtles may be partly due to the fact that terrapins are challenging to monitor because of their cryptic ecology and their small body size that limits the use of animal-borne automatic devices.

Estimating time-budget and energy expenditure in free living animals is a key step to understand the physiology and ecology of species, their function within their ecosystem and, ultimately, their capacity to adapt to the present and future environmental changes [8]. To achieve these goals, animal-borne data-loggers have proven to be appropriate tools because they can record biological and environmental parameters at frequencies compatible with animals’ behaviour. Accelerometers can provide high frequency, high resolution information on individual posture, behaviour, and activity patterns of animals [8, 9]. Furthermore, activity metrics recorded with these devices (e.g. ODBA/PDBA) can serve as a proxy of animal energy expenditure associated to specific behaviours [10–12].

In turtles, reproduction is energetically costly, as adult females come ashore to lay clutches of numerous eggs that fill the entire maternal body cavity at maturation [13–15]. Because of such volume constraints, female turtles are most likely to fast during the nesting season [16]. The European pond turtle, *Emys orbicularis* (Linnaeus, 1758), is a small-sized freshwater turtle that occurs throughout Europe, even reaching the Middle East [17]. It has been reported to show a latitudinal gradient in its reproductive effort [18], with potential double clutches under the warm Mediterranean climate [19]. Its basic behaviour and activity have been extensively investigated (mainly basking, during which animals exit water to increase body temperature via solar radiation [20–22], and displacements [23, 24]). Yet, the fine scale daily and seasonal behaviour patterns are almost unknown in the European pond turtle as in most freshwater turtles.

Given the above-mentioned ectothermic physiology and constraints related to reproduction both in terms of time and energy, the European pond turtle has been shown to adjust its behaviour and time-budget according to thermal conditions. Yet the actual changes in behaviour and energy expenditures throughout long periods of time, such as the entire nesting season, have not been reported yet. Using accelerometers combined with sensors measuring ambient temperature, pressure, and light, we investigated the fine scale behaviour and associated activity patterns of adult female European pond turtles living freely in semi-natural captive conditions in relation to meteorological conditions throughout the nesting season in Alsace, NE France. We predicted that activity patterns will be lower during the night, when compared with the day, due to diurnal fluctuations in air temperature, rainfall and/or cloud coverage, yet with maximum daily activity patterns around oviposition.

## Materials and methods

### Study area

The study was carried out in the conservatory captive breeding facility located at the research station of Petite Camargue Alsacienne (St Louis, Alsace, France; 47.63°N | 7.54°E), between April 13 and August 29, 2017. The facility consists of a 1200m^2^ outdoor enclosure, which includes one 250m^2^ artificial pond (< 2m depth) connected to the underground water table and surrounded by natural vegetation. It also contains an artificial mound facing South, where females can crawl for egg deposition. There, 22 (15 female and 7 male) pond turtles (*Emys orbicularis orbicularis*, captured in 2004 as adults of unknown age from a natural population in La Brenne, France) are kept as part of a conservation program, aiming at reintroducing the species in Alsace, NE of France [25]. Therefore, nesting activity is monitored from May to July by daily observations (from 6pm to 11pm) for recording individuals laying (or attempting to lay) eggs [26].

### Subjects and tools

This study focused on eight adult females European pond turtles (body mass: 749 ± 171 g [mean ± standard deviation], carapace length 155 ± 14 mm) equipped with dataloggers (hereafter referred as WACU) developed at IPHC (https://iphc.cnrs.fr). WACUs are miniaturized autonomous recorders (21 × 13 × 4 mm, 7 g including batteries and potting) measuring temperature (T_WACU_), pressure (P_WACU_) and light (L_WACU_) at 1 Hz and 3D acceleration at 10 Hz over 4 to 6 months (see general specifications for WACU on http://iphc.cnrs.fr/-MIBE-.html). The mass ratio between dataloggers and turtles (<1%) suggests that such deployments cause limited, if any, disturbance to the turtles.

WACUs were fixed directly on the top of the turtle carapace using fast-setting epoxy (Araldite) after epibiontes (organisms living on the carapace) and dead tissues were removed by successive and alternating applications of acetone and sand paper (sand grain 80). The entire attachment procedure lasted between 10 and 20 minutes depending on glue setting. Turtles were released ~1 hour after WACU attachment. Before attachment to a turtle and after retrieval, each WACU was rotated over all angles at exact GPS times, used as time stamps, to assess and correct the potential drift of their internal Real Time Clock (RTC) throughout the duration of deployment. All times are given as UTC (Universal Time Coordinated), while local time was UTC+2.

In addition to data recorded by the WACUs, air and water temperatures were recorded at the study site using Tinytags data-loggers (Gemini Data Loggers, Chichester, UK, https://www.geminidataloggers.com/fr/). These loggers recorded temperature every 10 minutes: (1) in the air (T_AirField_), (2) at three different depths within the water column of the pond, using three devices that were suspended at a vertical rope: (a) at the surface (T_WaterSurface_), (b) at 20 cm depth (T_Water20cm_), and (c) at the bottom of the pond (T_WaterBottom_). Finally, meteorological records from the weather station of the Bale-Mulhouse Airport (Météo-France station 07299, GPS position: 47.61°N | 7.51°E, 263m above sea level, 3km West from the field site) were obtained from the national weather service (https://www.meteofrance.fr/): this concerned (3) air temperature (T_AirMulhouse_), cloud cover, and rain fall, recorded every 3 hours. There was a significant positive correlation between air temperature at the study site and at the airport (T_AirField_ [°C] = 0.053[±0.135] + 1.014[±0.007] × T_AirMulhouse_ [°C], R^2^ = 0.865, p<10^−12^, n = 3324 hourly records). Sunrise and sunset times for the nearby city of Mulhouse (Alsace, NE France) were downloaded from https://www.timeanddate.com.

Animal handling was approved by the French Ministry for National Education, Higher Education and Research and by the Ethical Committee for Animal Experimentation (CREMEAS, CEEA 35, Strasbourg, APAFIS#649–201505121120811_v1).

### Data handling

Data were handled, pre-treated, visualized and analysed using Matlab (version 8.6.0.267246 [R2015b] August 20, 2015). Real Time Clock (RTC) drift was 21.30 ± 2.72 min over 141 days of functioning, range [18.40–27.10 min, n = 8 WACUs]. Recorded pressure (P_WACU_) was linearly transformed to indicate the depth under water surface (i.e. 0 at the surface, 0.1 bar at 1m depth). The 3-axis acceleration signal was decomposed using a low-pass filter (filtfilt Matlab function) following [8]. We used a 2 second window for the low pass filter to minimize variance of the vectorial sum of the static components. For each axis, the low-pass filter extracted the static (due to gravity) and dynamic (due to a change in velocity) components of the acceleration signal [8]. The Vectorial Dynamic Body Acceleration (VeDBA, in m.s^-2^) was calculated as the vectorial sum of the absolute values of the dynamic component of the three axes: in other words, VeDBA is the total acceleration due to changes in animal velocity. When VeDBA is integrated over a certain time window, the result (in m.s^-1^) can be used as a proxy of the animal’s locomotor activity. Angles of the anterior-posterior (pitch) and left-right (roll) axes in relation to the horizontal axis were computed from the static component of acceleration.

### Identification and analysis of daily patterns

**Fig 1:**
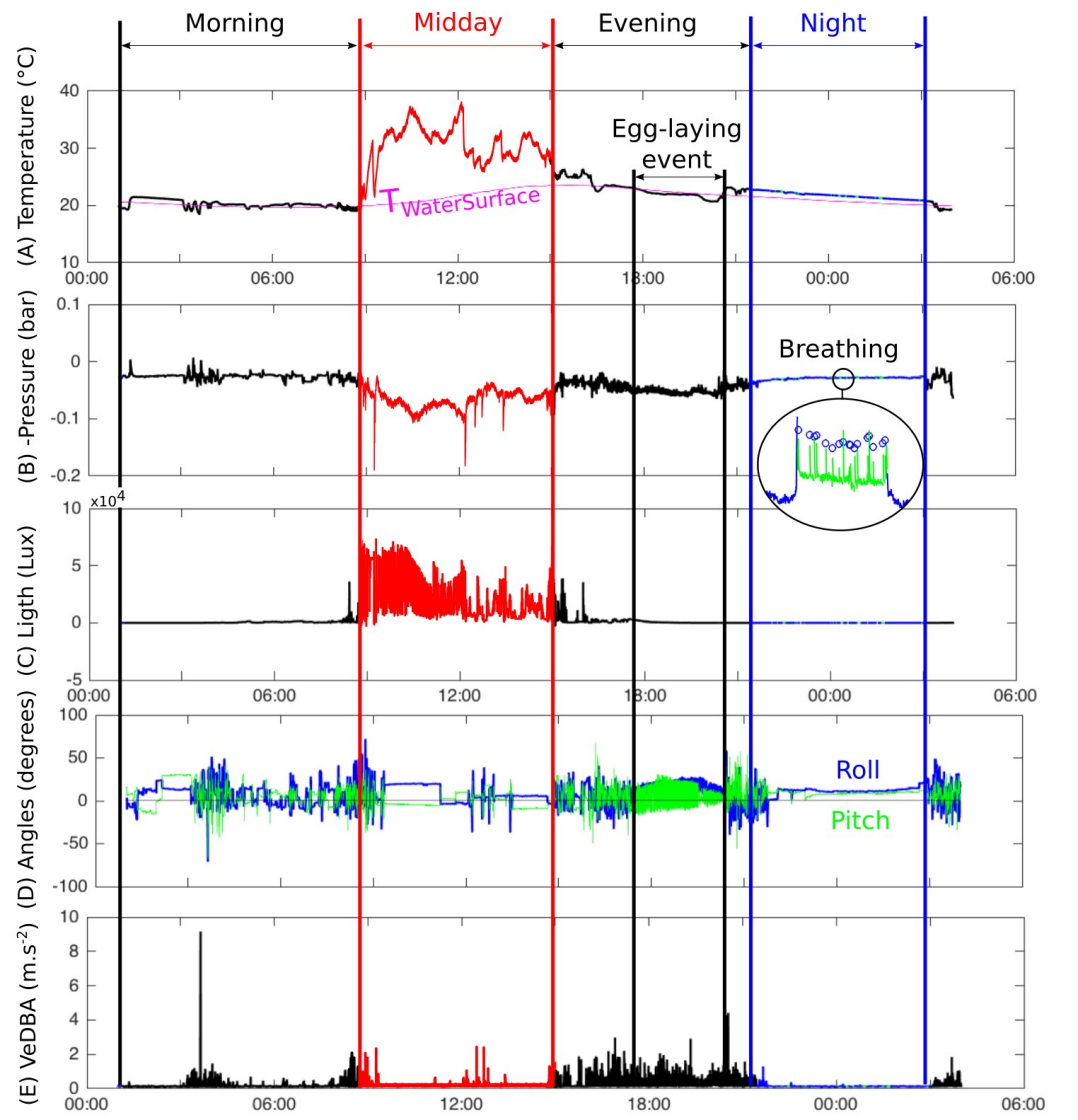
Example of a 24-h record collected by a WACU deployed on an adult female European pond turtle *Emys orbicularis* (individual 8, on May 31, 2017, UTC time) at Petite Camargue Alsacienne, Alsace, France, from which daily schedule could be estimated as Morning, Midday, Evening and Night bouts. From top to bottom: (A) Temperature: T_*WaterSurface*_ in pink and T_*WACU*_ in black for Morning and Evening, in red for Midday including basking, in blue for Night and in green for breath bouts during Night. (B) Pressure (reversed vertical axis) showing a breath bout in details, with blue circles corresponding to inhalation events. (C) Ambient Light (see T_*WACU*_ for colours). (D) Angles (in degrees) extracted from static components of acceleration from which egg-laying events can be inferred. (E) VeDBA from dynamic component of acceleration computed on a 1 min window.

As a first step, T_WACU_, P_WACU_ and L_WACU_ were used to investigate potential changes in associated patterns across the 24-hour cycle. This permitted to divide a 24h cycle in four phases (Fig 1):

1. “Night” was characterized by low light values (Median[L_WACU_]<1000 Lux calculated over a 1 min window) and highly consistent temperature and pressure readings (sd[T_WACU_]<1 & sd[P_WACU_]<5e^-3^ computed over a 1 min window). During Night, “Breathing” bouts could be identified by sudden changes in P_WACU_. Pressure spikes within Breathing bouts were defined as “Inhalation” events (Fig 1B).
2. “Midday” was characterized by high light values (Mean[L_WACU_]>20000 Lux over a 1 min window) and increased temperature values, i.e. several degrees higher than the lowest temperature of the 24-h-period. During Midday, “Basking” events were defined as periods where T_WACU_ continuously increased over time. Sudden drops in T_WACU_ or sudden increases in pressure were interpreted as “Diving” events.
3. “Morning” was defined as the period between Night and Midday.
4. “Evening” was defined as the period between Midday and Night.

To detect egg-laying events, we constructed an algorithm that could detect all laying events visually observed in the field. This algorithm was based on the angles extracted from the static component of acceleration. Egg-laying events were characterized by variance[roll]<50 and variance[pitch]>120 or variance[roll]<10 and variance[pitch]>40 computed over a 2 min window that fitted with the movements of rear legs of turtles digging their nest.

Apart from Night phase, P_WACU_ could not be used in all cases for depth assessment because of erratic pressure signal during active behaviours occurring the rest of the day (see Discussion below). To assess the depth of the animals in the water column (above, at the surface, at 20cm or more below the surface), we thus used correlations of T_WACU_ with all four temperatures recorded in the field with Tinytags. We reasoned that the best correlation between T_WACU_ and the temperature of the surrounding environment (Intercept ≈ 0, slope ≈ 1, R^2^ ≈ 1) would indicate at best the vertical position (in air or water) of the turtle (Fig 1A).

Statistical models were constructed in R (R version 3.2.4 [2016-03-10]), using the packages *nlme* for linear mixed models, and *lme4* for GLMMs (Generalized Linear Mixed Models [27, 28]). To model the probability of basking behaviour (i.e. during Midday), we ran a GLMM with a binomial distribution of Midday phases as variable. Fixed factors were environmental parameters (temperature, rain fall, cloud coverage, daylength at Mulhouse weather station) that were preliminarily centred and reduced (mean = 0 and variance = 1). Turtle ID was used as a random variable to deal with pseudo replication in the dataset [29–31]. Using a stepwise backward analysis based on AIC, we selected the most parsimonious model. The slope was considered as a proxy for the weight of the effect.

## Results

All eight WACUs provided complete records during their deployment, leading to 137 days of data for each individual, i.e. 1096 complete days in total.

### Daily activity patterns

(1) Over the entire records, Night phases lasted on average 9.3 ± 3.4 hours. Each Night phase contained on average 23.2 ± 14.3 Breathing bouts. One single Breathing bout lasted on average 162 ± 103 s and consisted of 9.6 ± 7.2 inhalation events (i.e. 222.0 ± 158.9 inhalation events per Night) (Table 1). Time elapsed between two successive Breathing bouts was 22.5 ± 76.9 min (90%-quantile = 40.5 min, n = 25550).

**Table 1:** Summary of individual biometrics, nesting event characteristics, daily bout durations and associated VeDBA in 8 adult female European pond turtles, *Emys orbicularis*, monitored at Petite Camargue Alsacienne, Alsace, France, from 14 April to 28 August, 2017.

Turtle ID #		1	2	3	4	5	6	7	8	**Total**
Body mass (g)		549	551	670	730	726	790	962	1015	**749±171**
Carapace length (mm)		135	138	150	155	161	155	169	177	**155±14**
Nb of nesting attempts/events detected by accelerometry		6	5	1	4	3	3	1	3	**3.25±1.75 (n = 26)**
Dates of nesting *detected by accelerometry		08 June	11 June	31 May	02 June	3 June* and 25 June*	02 June	02 June	31 May	**[31 May-25 June]**
Egg-laying events	Mean Duration (h)	1.72	1.11	1.00	0.98	1.41	0.70	1.00	1.20	**1.22±0.63**
	Mean Hourly VeDBA (m/s)	980.7	1098.3	914.2	1203.9	1251.5	1473.6	1118.2	833.7	**1111.7±224.6**
Morning	Duration (h)	6.14±3.52	6.63±4.35	5.80±3.96	7.41±4.06	6.30±4.02	6.94±4.46	6.59±4.17	6.08±3.34	**6.49±4.02**
	Hourly VeDBA (m/s)	607.1±142.0	645.8±143.7	698.3±135.1	635.4±155.1	669.3±138.8	600.4±122.6	577.3±99.5	555.7±87.4	**623.8±137.0**
	n	136	137	137	136	136	137	135	136	**1090**
Midday	Duration (h)	4.99±2.42	5.33±2.14	5.83±2.57	5.17±2.41	5.74±2.58	4.34±2.61	5.14±2.58	4.33±2.56	**5.11±2.53**
	Hourly VeDBA (m/s)	535.5±100.5	577.9±135.4	549.4±105.1	562.7±140.7	562.4±127.1	527.6±100.4	509.5±84.3	526.0±85.3	**543.6±113.1**
	n	117	118	121	114	119	115	125	120	**949**
Evening	Duration (h)	4.07±2.22	3.91±1.67	3.96±2.05	4.35±1.71	4.10±2.16	4.69±2.35	5.57±3.52	4.01±2.03	**4.34±2.34**
	Hourly VeDBA (m/s)	578.0±168.7	635.3±176.2	595.2±135.0	603.1±149.2	591.7±141.4	547.9±133.3	541.6±106.2	527.5±113.6	**577.2±145.4**
	n	117	118	121	114	119	115	124	120	**948**
Night	Duration (h)	10.09±3.47	9.33±3.15	9.49±3.32	8.66±2.96	9.14±3.47	9.47±3.52	7.77±3.42	10.58±3.4	**9.32±3.43**
	Hourly VeDBA (m/s)	417.6±6.3	412.1±8.6	444.7±8.7	408.8±19.4	412.0±9.1	417.3±7.1	430.6±11.5	430.0±7.1	**421.6±15.6**
	n	138	138	138	138	138	137	137	138	**1102**
Basking	Duration (h)	1.63±0.86	1.78±0.89	1.83±0.89	1.74±0.96	1.73±0.88	1.50±1.04	1.63±0.89	1.53±0.97	**1.67±0.93**
	n	117	118	121	114	119	115	125	120	**949**

Values are means ± sd.; n represents the number of events for each variable.

We manually checked three series of 24 continuous hours for four individuals (i.e. 12 complete 24-h cycles), chosen at random, to estimate the efficacy of our algorithm. In total, 262 Breathing bouts were correctly detected by the algorithm, 38 were false positives (12.7%) and 31 were false negatives (10.6%). 2795 Inhalation events were correctly detected by the algorithm, 484 were false positives (14.8%) and 425 were false negatives (13.2%). For both events, the number of false positives was close to number of false negatives. Therefore, we are confident that our algorithm estimated the actual number of Breathing bouts fairly accurately. Yet, the time required to manually analyse these 12 random 24-h cycles was 20,000 times greater than that needed when using the algorithm.

Mean P_WACU_[during Night phase] was 0.037 ± 0.016 bar, giving a mean depth at the top of the carapace of 37 ± 16 cm below the surface at night. Yet the temperature on contact with turtles (T_WACU_) during Night was best correlated with water temperature at the surface (T_WACU_[during Night bouts] = 0.53[±10^−3^] + 0.99[±<10^−3^] × T_WaterSurface_ [during Night bouts], R^2^ = 0.97, P < 10^−12^, n = 37e^6^), suggesting that turtles spent their night submerged close to the surface (see Fig 1A for example). This is confirmed by the calculation of the mean difference between the pressure recorded just before a breathing event, and the minimum pressure during a breathing event, that was 0.0044 ± 0.003 bar, indicating that at night, turtles rose on average 4.4 ± 3.1 cm out of the water when breathing.

(2) Midday phases did not occur every time: over the 1096 recorded 24h-cycles (one for each day and each turtle), 142 showed no Midday phase (Table 1). 78 of these events were common to at least seven of the eight turtles and concerned 10 given days. GLMM analysis showed that the probability of Midday phase to occur was best explained by cloud coverage (slope = -2.52[±0.28], z-test = -8.9, p < 2e-16), before DayLength (slope = 0.75[±0.12], z-test = 6.1, p = 1.e-9) and rain fall (slope = -0.35[±0.08], z-test = -4.3, p = 1.e-5).

(3) Morning phases were characterized by a specific moment of the day, when T_WACU_ was close to T_WaterSurface_ (R^2^ = 0.925), yet with highly variable Pressure readings (unlike Nights) (Fig 1). This indicates that turtles were spending their Morning submerged close to the surface, while being relatively active (as confirmed by the acceleration data, see below, Table 1).

(4) Evening phases were associated with slow linear decrease in T_WACU_, yet, with variable P_WACU_ (Table 1).

### Changes in activity levels throughout the nesting period

**Fig 2:**
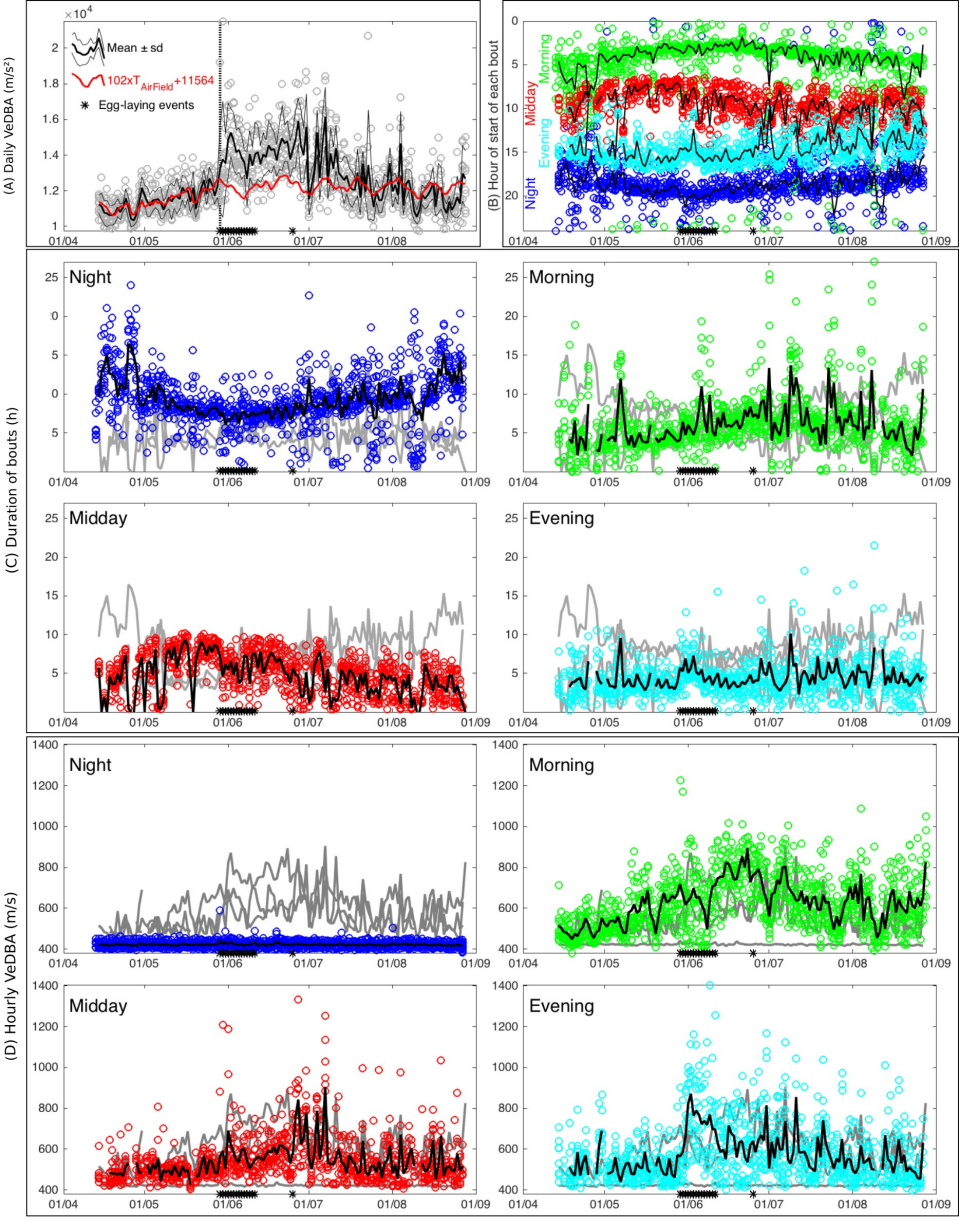
Time-budget of 8 adult females European pond turtle *Emys orbicularis* at Petite Camargue Alsacienne, Alsace, France from 14 April to 29 August, 2017. For each day and each individual, the sum of VeDBA computed over 24h is represented with a single circle. On all graphs, black asterisks on the x-axis represent egg-laying events detected by accelerometry signal. (A) Thick and thin lines are respectively the daily mean ± standard deviation computed for all 8 individuals. The red line is a linear transformation of air temperature recorded on the field (TAirField). Parameters were estimated by plotting TAirField against daily VeDBA over the period preceding egg-laying date. Note that this linear transformation fits well with mean VeDBA over this period (left from the vertical dotted line), but not after egg-laying date. (B) Hours of start and end of each daily bout, with means shown as solid lines. Hours of start of Morning, Midday, Evening and Night bouts are respectively in green, red, cyan and blue. (C) Duration of each bout, with black solid line for the daily mean. (D) Hourly VeDBA for each bout, with black solid line for the daily mean. At the far-right bottom graph, note the huge increase in hourly VeDBA during the evening bout when egg-laying occurred.

VeDBA was used to assess activity levels and showed three main periods relative to the egg-laying date. Daily VeDBA showed a large increase around the egg-laying date (Fig 2A), mostly due to increased Evening activity (Fig 2D), when egg-laying occurred. Before the egg-laying date, daily VeDBA was best explained by VeDBA computed during Morning phase (R^2^ = 0.61, p<2e-16) compared to the other phases. More precisely, daily VeDBA was best correlated with hourly VeDBA computed within the [4am-10am, UTC] window of the day (R^2^ = 0.76, p<2e-16). Before the egg-laying date, daily VeDBA was also positively correlated to T_AirField_ (R^2^ = 0.33, p <2e-16) (Fig 2A), whereas this relation was weaker afterwards (R^2^ = 0.06, p = 1e-10). VeDBA during Morning was also positively correlated to T_WaterSurface_ before the egg-laying date (R^2^ = 0.35, p<2e-16), but the relationship was weak for the rest of the season (R^2^ = 0.087, p = 5e-16). Hourly VeDBA was clearly influenced by air temperature through a non-linear pattern: maximal hourly VeDBA increased with air temperature until around 25°C but then decreased at higher air temperatures.

### Identification of nesting events

Nocturnal field patrolling (from 6pm to 11 pm from 15 May to 5 July, 2017) permitted to observe all but one (Turtle#5) of the eight turtles crawling on the mount for nesting. During digging nest, before a turtle deposits its eggs, the angles extracted from the static component of acceleration showed a very specific pattern, associated with regular, repetitive leg-movements (Fig 1D). This acceleration signal was sufficiently stereotypical to allow detection of nest digging by a computer algorithm (see Methods). Our customised algorithm detected all nesting observed in the field, but also digging that did not led to successful oviposition (nesting attempts), plus three additional nesting events and attempts for the Turtle#5 that had not been directly observed nesting in the field. All acceleration-derived nest digging and nesting events occurred between 29 May and 25 June, 2017, during the Evening. Only Turtle#5 that was not observed during nocturnal patrols nested during the Morning. On average, nest digging lasted 73 ± 38 min and was associated with a mean VeDBA of 0.31 ± 0.06 m.s^-2^ (Table 1). As a comparison, mean VeDBA computed over the entire dataset was 0.16 ± 0.12 m.s^-2^, i.e. half that observed during nest digging. Accordingly, nesting (walking, nest digging, laying eggs, nest covering) appears as a highly energy-consuming activity.

## Discussion

This study shows that 3-axis accelerometry associated with simple temperature, pressure, light sensors provide unrevealed fine-scale behaviours and time-budget in free living freshwater turtles throughout the nesting season. We show that in Alsace, NE of France, adult females behave according to four distinguishable phases over the circadian cycle, referred as Night, Morning, Midday, and Evening. Each of the four phases were associated with particular behaviours and activity levels. Additionally, the high resolution and high frequency data collected with these devices permitted to assess breathing events, timing of nesting events and energetic proxies (VeDBA) derived from acceleration. Importantly, we show that the nesting events appear to be a turning point in terms of individual behaviour, energy expenditure, and environmental drivers: before nesting occurs, the daily activity is highly related to ambient temperature, whereas it shows more stochastic patterns and changes afterward (Fig 2A and 2D).

### Automatic detection of daily patterns

The algorithm we developed to identify daily patterns has proven a powerful, fast and customisable tool. First, it allowed to work on extensive datasets (one 3-axis accelerometer operating at 10Hz over 140 days provides 130 million lines of [Time,X,Y,Z], i.e. ~4.5 GB of data), without subjectivity in pattern cutting. Second, despite automatic detection based on threshold values that may introduce biases (e.g. a point just above a threshold value is automatically removed whereas a visual/manual treatment may have led to take it into account), visual inspection of 12 randomly chosen days revealed that the algorithm is highly robust and consistent in terms of number of events.

### Foraging occurs in the morning

In the morning, turtles experienced temperatures (T_WACU_) closest to T_WaterSurface_ (Fig 1A), indicating that turtles mostly remained close to the surface, as confirmed by direct field observations. The subsurface layer has been proposed as the preferred environment for foraging in pond turtles [32, 33]. However, actual behaviour of WACU-equipped individuals monitored in our study was hard to deduce from recorded data only. There was neither an informative signal in the temperature records, nor a clear pattern in pressure and angle data. Yet, the pressure signal was very erratic, suggesting that turtles repeatedly banged into obstacles with the logger attached to the carapace. Such erratic pressure signal constrained us to use water temperature as a proxy of the water depth where turtles actually behaved. We suggest that erratic pressure signals could be associated with motivated intrusions of turtles in reeds where they may actively seek for food. Putative foraging during Morning is supported by high VeDBA but most importantly by the significant effect of ambient temperature between 4am and 10am on daily VeDBA: the warmest the water, the most active turtles were foraging. Previous studies reported feeding mostly occurs in morning in other Emydidae [34]. At the opposite, it is very unlikely that feeding occurs during Night because VeDBA was minimal at that time of the day and light limited for turtles visually foraging. In short, it is most likely that feeding occurred early in the day, before basking, with the latter facilitating digestion during warmer periods in the middle of the day. In the future, underwater or animal-borne cameras should be used to test our hypothesis that Morning is the most prone phase of the day for foraging in pond turtles.

### Nesting is a nightly energy-consuming activity

25 of the 26 detected nesting events and attempts occurred between May 29 and June 11, 2017, and all but two occurred during the Evening. These nesting dates and their timing are in line with the literature for France where 76% (n = 21) of *Emys* turtles nest after sunset, with the remaining nesting in the morning [24]. *Emys* turtles have been reported to nest between the end of May and the end of June in France [35] and in Turkey [36], and throughout June in Italy [37]. Interestingly, one of our eight individuals (ID#5, Table 1) laid two successive clutches 22 days apart. Double (and even triple) clutches have been reported in *Emys orbicularis* [19] but is thought to be quite rare in France [35]. Temperature but also sunshine duration have been reported to be important factors influencing nesting: in Slovakia first nesting attempts occur after a 13-day period when temperature is 17.7°C [38]. We found similar values, with mean T_AirField_ being 18.6°C during the same 13-day period.

Our results show that in our captive facility where the nesting mount was only a few meters from the pond, nesting events and attempts are highly energy-consuming activities, about twice more expensive in terms of VeDBA than any other activity recorded in the present study (Table 1). Importantly, all eight studied individuals made 3.25 ± 1.75 attempts before they successfully laid eggs (eventually laying twice, as reported for ID#5), increasing by three times the actual energy and time devoted to nesting. In natural conditions where *Emys* turtles can travel 150 to 1000 m to their nesting ground [39], actual energetic expenses are most likely to be even more extended. Deployment of accelerometers on free ranging turtles are required to estimate such costs in the wild.

In addition, nesting appears as a turning point in the season. Before it occurs, turtles are mainly active in the morning (Fig 2D), probably foraging. The level of activity is then closely linked to the ambient temperature (Fig 2A). During the nesting period, daily activity doubles (Fig 2A), due to the intense nest digging activity happening in the evening (Fig 2D). After then, turtles are more active during the evening and the midday time (Fig 2D), and activity is no more linearly linked to ambient temperature (Fig 2A).

### Temperature has a dual effect on the level of activity

We noticed an interesting non-linear effect of ambient temperature on the hourly activity level (VeDBA), with a turning point around 25°C. VeDBA having a Gamma-like distribution, it is convenient to focus on maximal VeDBA values: for temperatures between 7 and 25°C, maximal VeDBA increased with temperature experienced by the turtles, whereas maximal VeDBA decreased when temperature exceeded 25°C (until the maximal air temperature recorded in the study, i.e. 47°C). These results suggest that for temperatures lower than 25°C, European pond turtle activity is positively related to temperature. Above this temperature threshold, which typically occurs during the early afternoon, individuals are most likely to bask or rest, leading to lower activity: the warmer the air, the more turtles lay immobile emerged. Turtles were least active during the Night. However, we did not detect any bimodal activity patterns during daytime (as described in [40]).

Air temperature and sunlight have been previously reported to drive basking behaviour in freshwater turtles [24, 41]. Our study showed that in Alsace, NE France, cloud coverage rather than temperature most strongly affected the basking behaviour of turtles during Midday. For instance, a cloud coverage > 90% dramatically reduced the number of basking events, if they occurred at all. We propose that when the sun is not visible, individuals rather stay submerged, since basking would be ineffective, unnecessarily elevating potential predation risks. Recording the level of solar radiation experienced by the turtles directly might even better explain observed basking behaviour, as clouds are not uniformly obstructing the sun.

## Conclusion

Accelerometers deployed on gravid *Emys* turtles permit to assess fine scale behaviour, time budget and energetic expenditure, and most interestingly to quantify nesting events, which are likely underestimated in the field, because they mostly occur during the night. The nesting period appears to be the most intense activity in terms of energy expenditure that needs to be estimated in natural conditions. Nesting also happens as a turning point for seasonal activity patterns: before nesting, daily activity is mainly due to foraging-like, temperature-driven activity in the Morning, whereas after nesting, individual activity is more complex. In the near future, video-assisted monitoring of turtle behaviour and calibration of actual energy expenditure with accelerometry-derived VeDBA, coupled with spatial telemetry should permit the extension of time budgets to quantitative energy budgets.

Our study may have some limitations, mainly due to the fact that all individuals inhabited one single, relatively small pond, where all individuals encountered the same environmental constraints. One may, thus, stress that each individual’s behaviour may have impacted that of the rest of the group. For instance, some turtles (*Pseudemys nelsoni*) have been reported to present social learning by observing conspecifics [42]. In the future, social structure should be investigated for assessing such potential inter-individual interactions.

## Abbreviations

VeDBA: Vectorial Dynamic Body Acceleration
T_AirField_: Temperature of air, recorded on the field
T_WaterSurface_, T_Water20cm_, T_WaterBottom_: Water Temperature recorded at the surface, 20 cm below the surface, and at the bottom of the pond, respectively
T_AirMulhouse_: Temperature of air recorded in Mulhouse airport, by Météo France
T_WACU_, P_WACU_, L_WACU_: Temperature, Pressure and Light recorded by dataloggers attached on the carapace of turtles; these are not internal measurements
g: grams
m: meters
°C: Celsius degrees
min: minutes
s: seconds
sd: standard deviation
